# Electronic nose dataset for beef quality monitoring in uncontrolled ambient conditions

**DOI:** 10.1016/j.dib.2018.11.091

**Published:** 2018-11-22

**Authors:** Dedy Rahman Wijaya, Riyanarto Sarno, Enny Zulaika

**Affiliations:** a Informatics Department, Institut Teknologi Sepuluh Nopember, Jalan Raya ITS, Keputih, Sukolilo, 60111 Surabaya, East Java, Indonesia; b School of Applied Science, Telkom University, Jalan Telekomunikasi, Terusan Buah Batu, 40257 Bandung, West Java, Indonesia; c Biology Department, Institut Teknologi Sepuluh Nopember, Jalan Raya ITS, Keputih, Sukolilo, 60111 Surabaya, East Java, Indonesia

## Abstract

In recent years, the development of a rapid, simple, and low-cost meat assessment system using an electronic nose (e-nose) has been the concern of researchers. In this data article, we provide a time series dataset that was obtained from a beef quality monitoring experiment using an e-nose in uncontrolled ambient conditions. The availability of this dataset will enable discussion on how to deal with noisy e-nose signals and non-optimum sensor array in beef quality monitoring. Hence, the development of proper signal processing and robust machine learning algorithm are several challenges that must be faced. Furthermore, this dataset can also be useful as a comparison dataset for similar e-nose applications, such as air quality monitoring, smart packaging system, and food quality monitoring.

**Specifications table**

**Table:** 

Subject area	*Signal Processing, Food Science.*
More specific subject area	*Beef quality monitoring using an electronic nose.*
Type of data	*Table.*
How data were acquired	*The beef sample was monitored using a custom e-nose for 36 h.*
Data format	*Raw data, time series data.*
Experimental factors	*500 g of fresh extra-lean beef was tested using an e-nose in uncontrolled ambient conditions.*
Experimental features	*Nine features corresponding to nine metal-oxide semiconductor gas sensors. The discrete and continuous labels represent beef quality and microbial population in the beef sample, respectively.*
Data source location	*Surabaya, Indonesia.*
Data accessibility	*Dataset is available in Mendeley Data: https://data.mendeley.com/datasets/mwmhh766fc*
Related research articles	[1] D.R. Wijaya, R. Sarno, E. Zulaika, Information Quality Ratio as a novel metric for mother wavelet selection, Chemometrics and Intelligent Laboratory Systems, 160, 2017. doi:10.1016/j.chemolab.2016.11.012.
	[2] D.R. Wijaya, R. Sarno, E. Zulaika, S.I. Sabila, Development of mobile electronic nose for beef quality monitoring, in: 4th Information Systems International Conference 2017, ISICO 2017, Procedia Computer Science, Elsevier B.V., Bali, 2017, pp. 728–735. doi:10.1016/j.procs.2017.12.211.
	[3] D.R. Wijaya, R. Sarno, E. Zulaika, Sensor Array Optimization for Mobile Electronic Nose: Wavelet Transform and Filter Based Feature Selection Approach, International Review on Computers and Software, 11, 2016, pp. 659–671. https://doi.org/10.15866/irecos.v11i8.9425.

**Value of the data**

• The dataset is available as a reference for e-nose signal processing, notably for meat quality studies. The two main objectives of this dataset are multiclass beef classification and microbial population prediction by regression.
• This dataset will be useful for comparison purposes in other studies related to e-nose applications, including but not limited to food quality monitoring, intelligent packaging system, and air quality monitoring.
• The availability of this dataset will enable further discussion on e-nose signal processing, including how to deal with the noisy signals and the problem of overlapping selectivity in a sensor array.

## 1 Data

**Fig. 1:**
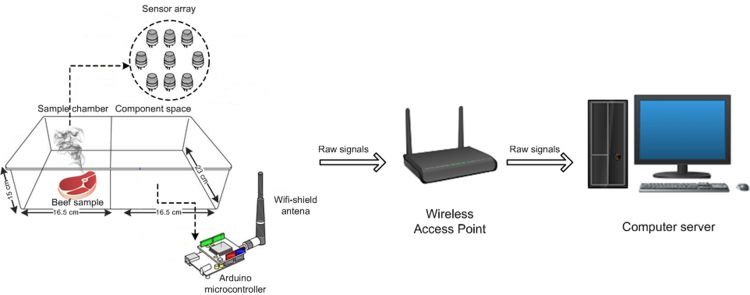
Schematic of the experimental design to acquire time series data from e-nose.

**Fig. 2:**
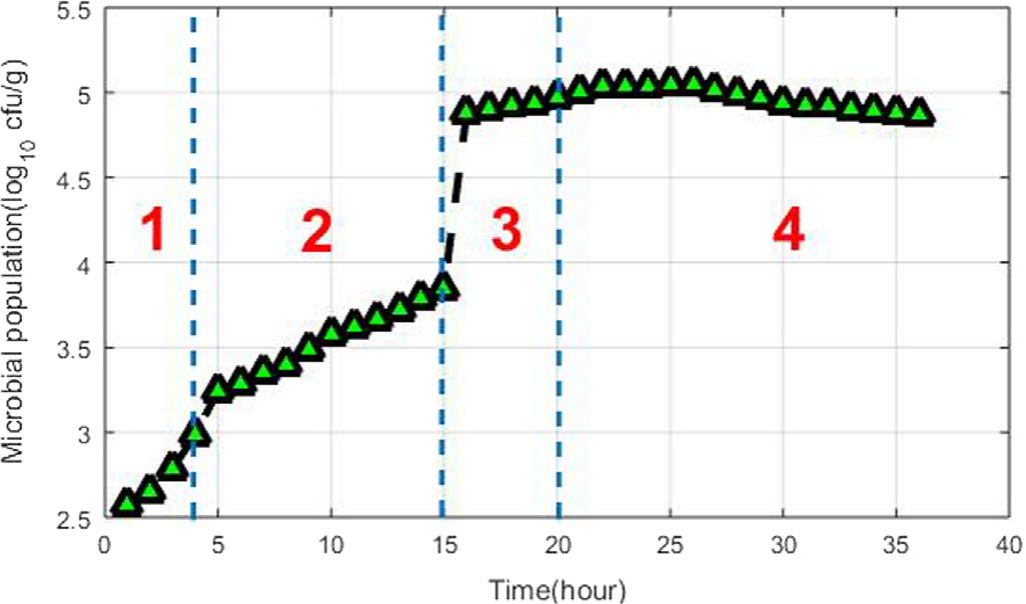
Microbial population: areas 1, 2, 3, and 4 indicate ‘excellent’, ‘good’, ‘acceptable’, and ‘spoiled’, respectively.

**Fig. 3:**
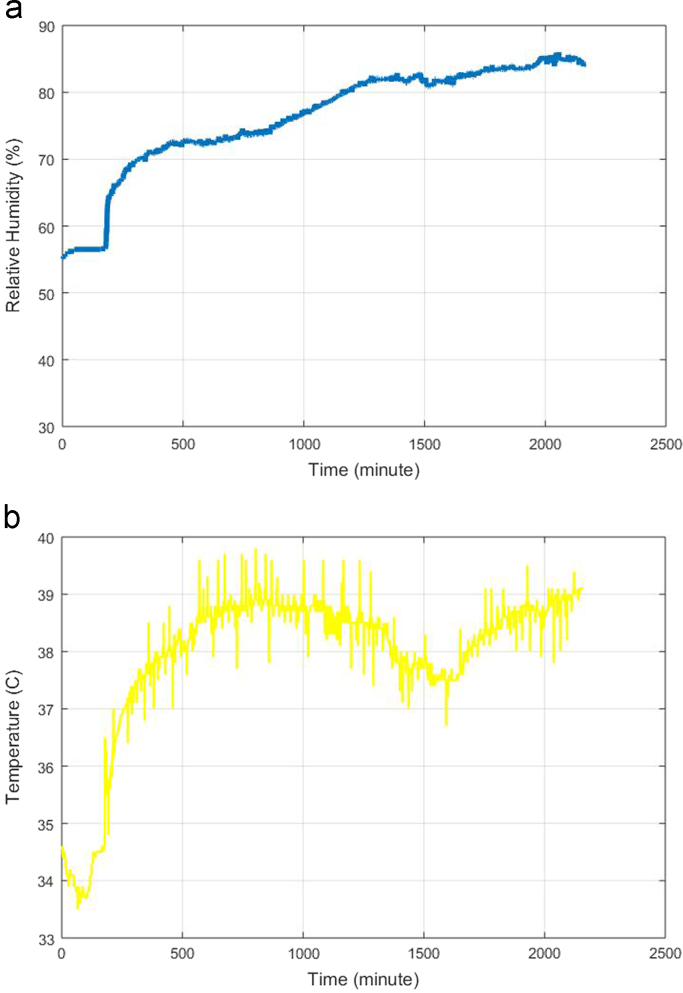
Changes in ambient conditions: (a) humidity and (b) temperature.

**Fig. 4:**
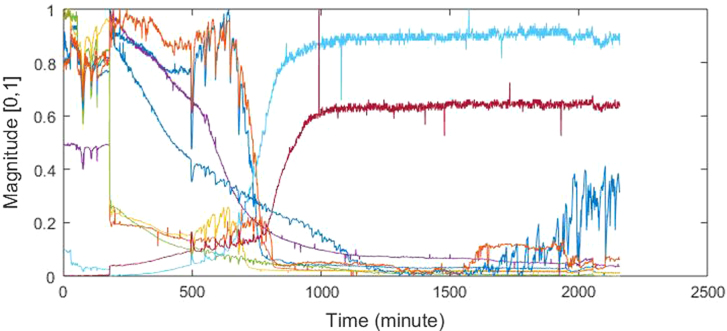
Example plot of normalized signal.

Table 1 lists the sensors used in this experiment. Fig. 1 shows a schematic of the experimental design. The sensory classes of beef are described in Table 2 and an example of the ground truth data is shown in Fig. 2. Fig. 3 shows an example of the changing ambient conditions measured during the experiment. A sample of the e-nose signals is depicted by Fig. 4. The dataset consists of five recorded time series, corresponding to five beef cuts, where one series contains 2160 min of measurement points. Every time series is distributed in comma-separated value format (csv). The first row contains the following column headers:

• Minute: time of measurement point (minutes);
• Class: discrete label of beef quality (‘excellent’, ‘good’, ‘acceptable’, ‘spoiled’);
• TVC: continuous label of microbial population (log_10_ cfu/g);
• MQ_: sensor resistance value of a particular gas sensor (Ω);
• Temperature: temperature (°C) in the sample chamber;
• Humidity: relative humidity (%) in the sample chamber.

**Table 1:** List of gas sensors.

No.	Gas sensor	Selectivity
1	MQ135	Carbon dioxide (CO_2_), ammonia (NH_3_), NO_x_, alcohol, benzene, smoke
2	MQ136	Hydrogen sulfide (H_2_S)
3	MQ2	Liquefied petroleum gas (LPG), i-butane, propane, methane, alcohol, hydrogen, smoke
4	MQ3	Methane (CH_4_), hexane, LPG, CO, alcohol, benzene
5	MQ4	Methane (CH_4_), natural gas
6	MQ5	LPG, natural gas, town gas
7	MQ6	Propane, LPG, iso-butane
8	MQ8	Hydrogen (H_2_)
9	MQ9	Propane, methane, CO
10	DHT22	Temperature, humidity

**Table 2:** Beef quality standard.

Class	Total viable count (log_10_ cfu/g)
Excellent	<3
Good	3–4
Acceptable	4–5
Spoiled	>5

^*^cfu/g: colony forming unit of bacteria in 1 g of meat.

The dataset is sorted in ascending order by the ‘Minute’ column. The experimental dates for each time series are as follows:

• Time series 1 (TS1.csv): May 12, 2016 to May 13, 2016;
• Time series 2 (TS2.csv): October 8, 2018 to October 9, 2018;
• Time series 3 (TS3.csv): October 11, 2018 to October 12, 2018;
• Time series 4 (TS4.csv): October 13, 2018 to October 14, 2018;
• Time series 5 (TS5.csv): October 15, 2018 to October 16, 2018.

## 2 Experimental design, materials and methods

### 2.1 Materials

An experiment was conducted to get time series data from beef quality monitoring using an e-nose. Signals from the prototype of a mobile e-nose were recorded. The device was built using an Arduino Mega ADK microcontroller and was equipped with a number of gas sensors, a temperature and humidity sensor, and a WiFi-shield. Metal-oxide semiconductor (MOS) gas sensors were assembled to build a gas sensor array. This type of sensor (hot sensor) was used because it is more resistant to moisture than cold sensors, such as surface acoustic wave (SAW), conductive polymer (CP), and mass acoustic wave (BAW) sensors [1]. The sensors used in the device are listed in Table 1. In this experiment, MQ gas sensors from Zhengzhou Winsen Electronics Technology Co., Ltd. were used. Their components consist of a tin dioxide (SnO_2_) sensitive layer, a micro AL_2_O_3_ ceramic tube, a measuring electrode, a heater fixed into a crust made of plastic, and stainless steel net. The heater provides the working conditions needed for the sensitive components [2]. The experiment was performed using 500 g of fresh beef from the market. To ensure that the meat was fresh, it was bought as early in the day as possible and information on when the cow was slaughtered was obtained from the seller.

### 2.2 Experimental design

Fig. 1 shows a schematic of the experiment. The sensor box was partitioned into two parts: a sample chamber and a component space. The sample chamber contained the sensor array, which was placed at the top of the sample chamber. In addition, a microcontroller and a WiFi-shield were placed in the component space to avoid damage due to exposure to gas and high humidity. The sensor box casing was made from a transparent acrylic material to make it easier to observe the sample׳s condition. The beef sample was placed beneath the sensor array in the sample chamber to accelerate odor detection. During the experiment, the sample chamber was almost completely closed off so that the airflow was minimal. This aimed to simulate the conditions when the e-nose is placed in a meat chiller or storage box. The response of the gas sensors is susceptible to be affected by changes in humidity caused by the production of water vapor from the process of meat decay. This happens because the sensor array and the meat sample are in the same space. Two identical slices of meat were used, where one slice was tested using the e-nose module and one was used for microbial analysis. Hence, the continuous monitoring using the e-nose was not interrupted by the bacterial analysis process.

The data were sent to a computer server at an interval of 1 min via a wireless network. The data were received by the computer server through self-developed middleware based on the TCP/IP protocol and immediately inserted into a MySQL database. The sensor resistance (Rs) was saved according to the type of resistive sensor used. The sensor resistance was calculated as follows:

(1) $$\mathrm{Rs}=\frac{\mathrm{Vc}-V_{\mathrm{RL}}}{V_{\mathrm{RL}}}\times \mathrm{RL}$$

(2) $$V_{\mathrm{RL}}=\frac{\mathrm{ADC}\times \mathrm{Vc}}{1023}$$

where $\mathrm{Vc},V_{\mathrm{RL}},\mathrm{RL},\mathrm{ADC}$ are standard voltage of microcontroller (5 V), current voltage, sensor load resistance, and ADC value, respectively. The Rs value changes when the gas sensor is exposed to a certain gas in the selectivity of the sensor.

The beef sample was continuously monitored for 36 h so the measurements were done in unstable conditions associated with the ambient temperature and humidity during day and night. Also, the process of beef spoilage generates water vapor that affects the humidity level in the sample chamber. External factors such as temperature and humidity can decrease sensor sensitivity, which is unfavorable for measurement repeatability [3]. In addition, a recent study indicated that ambient air variations are the most influential factor in MOS gas sensor stability [4]. Gas sensors are also susceptible to sensor poisoning caused by sulphur and ethanol compounds produced by protein decomposition [5]. This will occur during the beef monitoring process. Hence, the presence of noise in the signal is inevitable and is a major challenge in e-nose signal processing [6]. Another challenge is the existence of overlapping selectivity in the sensor array. This is the result of a non-optimal combination of sensors, which leads to performance degradation in the pattern recognition system, waste of electricity due to redundant sensor usage, and increased data size [7]. Thus, the dataset presented here is useful for comparison purposes in the utilization of e-nose applications for monitoring, especially for meat quality monitoring [8].

The meat standard from the Agricultural and Resource Management Council of Australia and New Zealand was used to determine the quality of the beef used in this experiment [9]. The quality of meat is divided into four classes based on the total viable count (TVC), as shown in Table 2. To acquire the ground truth data, a spectrophotometer with 1000× dilution was employed to measure the optical density. Furthermore, the microbial population was estimated using a hemocytometer. The procedure was based on a combination of the classical and the two-hour method [10]. Fig. 2 shows four areas, which correspond to four sensory classes based on the microbial population; the class (discrete) labels refer to the ground truth data. As can be seen, after 15 h the bacterial growth entered the exponential phase, characterized by a high growth rate.

Fig. 3 shows an example of the changes in temperature and humidity in the sample chamber during the experiment. The initial relative humidity was 55.1%, as shown in Fig. 3(a). The increase in humidity was caused by the process of beef spoilage, which generates water vapor. Moreover, the initial temperature was 33.5 °C. The experiment began in the morning at lower ambient temperature. The ambient temperature got higher towards noon and a further rise in temperature was caused by the gas sensors generating heat. Fig. 3(b) shows the temperature fluctuation during the day and the night. These uncontrolled ambient conditions affected the sensor responses as shown in Fig. 4. Thus, the e-nose signal was contaminated by external noise from uncontrolled ambient conditions.
